# Birth preparedness, complication readiness and associated factors among pregnant women in Agnuak zone, Southwest Ethiopia: a community based comparative cross-sectional study

**DOI:** 10.1186/s12884-020-2766-9

**Published:** 2020-02-03

**Authors:** Fikru Letose, Bitiya Admassu, Gurmesa Tura

**Affiliations:** 1 Gambella Peoples’ Regional National State Health Bureau, PO Box 109, Gambella, Ethiopia; 2 Department of Population and Family Health, Faculty of Health, Jimma University, Jimma, Ethiopia

**Keywords:** Birth preparedness, Complication readiness, Comparative cross-sectional study, Agnuak zone, Ethiopia

## Abstract

**Background:**

Birth Preparedness and Complication Readiness interventions have a significant role in the reduction of maternal and neonatal mortality risk. Inadequacy of birth and emergency preparedness were depicted as one of the major reasons for high maternal deaths in sub-Saharan Africa. The main objective of this study was to assess birth preparedness, complication readiness and associated factors among pregnant women.

**Methods:**

A community based comparative cross-sectional study design was conducted among 411 urban and 209 rural respondents who were selected using multi-stage stratified random sampling technique. Quantitative data were collected by interviewer administered questionnaire while qualitative data were collected from purposely selected 54 members of the community by using guiding checklist and analyzed by thematic areas. Birth preparedness and complication readiness was measured using five birth preparedness and complication readiness items then women who scored at least three were considered as well prepared. Bivariate and multivariable logistic regressions were used to examine the association between independent variables and birth preparedness and complication readiness. The result were presented as Odds Ratio at 95% CI. *P* < 0.05 ware used to dictate statistical significance.

**Results:**

A total response rate of the study was 97.3%. The prevalence of birth preparedness and complication readiness was significantly higher among urban respondents (*P* = 25.8%; *p* < 0.05). Factors such as history of obstetric complication, knowledge of key danger signs, having favourable attitude towards birth preparedness and complication readiness, starting antenatal care visit within 3 months age of pregnancy, completing at least four antenatal care visits, urban residence, having occupation of government employee or merchant and being in the higher wealth quintile were variables positively associated with birth preparedness and complication readiness.

**Conclusions:**

Prevalence of birth preparedness and complication readiness was low in this study, though significantly higher in urban area. Three-fourth of women planned to attend 4+ antenatal care visits indicating opportunity to counsel them on birth preparedness and complication readiness which increases its prevalence. Health workers should counsel every woman on birth preparedness and complication readiness components during her first antenatal care visit and subsequent visits.

## Background

Globally, 303,000 women die each year due to pregnancy-related causes. From these maternal deaths, high income countries have the lowest number which accounts approximately less than 1%. Contrary, Low and Middle Income Countries (LMIC) have about 99%, with sub-Saharan Africa (SSA) alone accounting for 66% of maternal deaths. Thus, maternal mortality remains the major public health challenge particularly in LMIC [1]. A woman from SSA has lifetime risk of death estimated at 1 in 36 contrasting sharply with 1 in 4900 in high income countries [1].

In Ethiopia, maternal mortality ratio reported was 412 deaths per 100,000 live births in 2016 [2]. However, this figure is still nearly two times higher than the country’s Health Sector Development Plan target to reduce maternal mortality rate to 267 maternal deaths per 100,000 live births by 2015 [3]. Developing and implementing Birth Preparedness and Complication Readiness (BP and CR) is one of the effective method to reduce this high maternal and neonatal deaths [4].

Birth Preparedness is a series of steps through which a pregnant woman and her family members are delivered with basic messages associated with pregnancy and giving birth to safeguard a healthy outcome for both mother and baby [5]. Since, all women and newborns are at risk of developing complication, and most of these complications cannot be predicted, the plan includes complication readiness to ensure an appropriate and timely response to any complication that may arise [6].

A BP and CR plan at individual level includes identification of the following components by the pregnant woman; preferred birth attendant, closest facility for appropriate health care, saved money for birth related and emergency obstetric expenses, arranging transport to a health facility for the normal birth and in the case of emergency, and identification of compatible blood donors [4, 7, 8].

A BP and CR interventions to reduce maternal and neonatal mortality in low-income countries showed a significant reduction of maternal and neonatal mortality risk by 53 and 24% respectively [9]. Thus, key messages of birth preparedness should be provided for a pregnant woman during antenatal care (ANC) visits such as every woman during labour and child birth should be managed by skilled provider, prepared for clean delivery and identified a person to accompany her during child birth [5]. Moreover, it is essential to all women and families to equip with adequate information about obstetric danger signs and what actions should be taken when an emergency occurs [4].

Community resources that sensitize and empower access to maternal health services also result in a rise in health facility childbirth and skilled birth attendance in rural SSA [10]. However, availability, accessibility, and affordability as well as the nature of public health care services and demand factors appear to contribute to the larger urban-rural inequalities on maternal health care [11].

A pregnant woman prepared for birth and knows obstetric danger signs encouraged her for utilization of skilled care [12]. Early booking and regular attendance to ANC in the course of pregnancy gives an opportunity for a woman to be counselled and make an appropriate plan for childbirth [13]. In this regard, about 34.8% of women from rural and 80.3% of women from urban area received ANC from a skilled person on midwifery services [14].

World Health Organization indicated that birth preparedness reduces home deliveries with a consequent increase in skilled attendance during labour and childbirth [15]. As stated in the Ethiopia Demographic and Health Survey (EDHS) report in 2016, rural women (19.7%) were less likely to deliver at health facility compared with urban women (72.9%). Moreover, proportion of women who received postnatal checkup within 2 days of childbirth were lower in rural area (12.6%) than urban area (45.2%) [2].

BP and CR may differ among pregnant women largely with regards to the level of development of the areas in which these women live. Moreover, quality of health service delivery were inefficient and usually poor in rural area than urban [3]. In SSA including Ethiopia, several studies showed low status of BP and CR [16–20]. However, this problem has not been assessed focusing on possible rural-urban differences as determinants of BP and CR. Moreover, studies conducted to assess BP and CR were limited to the other parts of Ethiopia. This study therefore conducted to assess the status of BP and CR among pregnant women and its associated factors in urban and rural area of Agnuak zone, Gambella Peoples’ National Regional State, Ethiopia. It is hoped that the results of the study will be used for priority setting and designing effective program to improve maternal and neonatal health.

## Methods and materials

### Study area and period

The study was conducted in Agnuak Zone from March 10 to April 10, 2017. Agnuak Zone is one of the three zones of Gambella Peoples’ National Regional state bordered in Southwest by South Sudan, Southeast by Southern Nations, Nationalities, and People’s Region (SNNPR), East by Mezhenger Zone, Northeast by the Oromia Region, and Northwest by South Sudan and Nuer Zone. Abobo town which is capital city of Agnuak Zone located at 822 km from Addis Ababa (the capital city of Ethiopia) and 45 km from Gambella town, capital city of Gambella Peoples’ National Regional state.

Agnuak Zone has five Districts (Woredas) namely Abobo, Dima, Gambella Zuria, Jor, and Gog which constitutes 21 urban and 59 rural kebeles (the smallest administrative unit). In 2017, the Zone has a total population of 158,875 from which 99,831 are urban residents. Pregnant women constitute 4766 from which 2995 were urban residents during the study period [21]. The Zone constitutes 21 urban and 43 rural health posts, 6 urban and 5 rural health centers, 6 urban private clinics, and 1 hospital. Currently, the zone has 5 ambulances [22].

### Study design and population

A mixed method of community-based comparative cross-sectional study design supplemented with qualitative data collection method was used. The quantitative study was conducted among randomly sampled urban and rural pregnant women who fulfill the inclusion criteria. The inclusion criteria for quantitative study were all pregnant women who were pregnant during study period for at least 3 months of age and residents in the sampled kebeles for more than 6 months. Pregnancy was confirmed by pregnancy screening criteria adapted from Stanback et al., 1999. Qualitative study was conducted among purposely selected pregnant women, health development army (HDA) leaders, and fathers.

In this study, critically ill pregnant women and those who unable to respond to the interview were excluded. Pregnant women and HDA leaders who were participated in the quantitative study were also excluded from Focus Group Discussion (FGD).

### Sample size determination and sampling technique

#### Sample size determination

##### For quantitative study

Two population proportion formula for comparative cross-sectional study design calculated by epi-info version 7.1.1.14 software was used to calculate the sample size. Different variables associated with BP and CR were checked to determine the variable which yields the largest sample. Among those variables checked for, attitude of women towards BP and CR yielded the largest sample size. Accordingly, the study conducted in Southwest Ethiopia [19] showed that prevalence of BP and CR among mothers having favourable attitude were 41.1% and having unfavourable attitude were 23.9%. The 95% CI and 80% Power were considered; Ratio of 2:1 was taken because pregnant women in urban and rural setting of Agnuak Zone were 2995 and 1771 [21] and also total pregnant women identified before actual data collection were 573 and 276 respectively; Design effect of 2 taken; and 10% was added for non-responses. Finally calculated sample size become n_1_ = 411 and n_2_ = 209.

##### For qualitative study

A total of six sessions of FGDs were planned to be conducted from which 3 in urban and 3 in rural area. Each FGD composed of 9 pregnant women, 9 HDA leaders and 9 fathers.

#### Sampling techniques

##### For quantitative study

Multistage stratified random sampling technique was used to select the study participants. In the first stage from the five districts of Agnuak Zone, three districts namely Gambella Zuriya, Abobo and Gog were selected by simple random sampling (SRS) method; lottery method. Each selected districts were stratified into urban and rural kebeles based on residence. In the second stage, 7 urban kebeles were selected proportionally from urban kebeles of the three districts by SRS; lottery method. In the same way, 10 rural kebeles were selected proportionally from rural kebeles of the three districts by SRS; lottery method. After study kebeles were selected, a list of pregnant women was obtained from family folder assisted by grade 10 complete and above females. Then, identification number was assigned for each pregnant woman who fulfilled eligibility criteria. Four hundred eleven and 209 pregnant women were proportionally allocated for randomly selected kebeles from urban and rural area respectively. At the end, SRS method (computer generated random numbers) was used to select those eligible pregnant women from family folder in each sampled kebeles.

Health Post is a two room structure of the most peripheral health care unit and the first level to deliver health care services for the community. Family folder is a paper based simple book in which all family members in their catchment health post were registered and updated every month. It is kept at the health post and used during visits to the family for provision and follow-up of health services they are receiving such as ANC or immunization.

##### For qualitative study

Purposive sampling technique was used to select the study participants. Accordingly, two kebeles namely Abaru from urban and Village 8 and 9 from rural area of Abobo district were selected purposely. These kebeles were carefully chosen due to their abundant population that might offer a possibility to get variety of participants. Then, a total of 9 pregnant women, 9 HDA leaders and 9 fathers each from the two kebeles were recruited for FGD. Those who are active participators during community affairs and expected to be participated actively during FGD were included in the study. The recruitment of these study participants were assisted by their kebele administrators.

#### Data collection instruments and procedures

##### Quantitative part

One female data collector per kebele assigned for each urban and rural area within each district. All data collectors were grade 10 and above who were living in the study area and fluent in speaking Amharic and Agnua language. For each district, one supervisor assigned to monitor entire data collection process. All supervisors were Bachelor of Science degree (BSc) holders with background on health profession. The whole data collection process were monitored by the principal investigator.

All data collectors identified eligible pregnant women by using their list from updated family folder and specific code marked on the list before actual data collection. In addition to this, a pregnant woman was identified by her district, residence (urban or rural), name of the kebele, Got name or number, and name of head of household.

Using a pre-tested questionnaire, information was collected through face to face interview. Socio-economic and demographic characteristics such as age, ethnicity, marital status, family size, residence, religion, education, household’s wealth assets and average time taken to near health facility on foot were included in the questionnaire. Danger signs during pregnancy, labour and childbirth and postnatal period were asked waiting their spontaneous response. A woman was also asked for number of ANC visits she planned to attend including currently attended, trimester of her pregnancy during the 1st ANC visit, history of previous pregnancy complication and attitudes and perceptions on BP and CR practice. Furthermore, a woman was asked for BP and CR practice waiting her spontaneous answer to check whether she was planned or practiced those operationally defined BP and CR component (Additional file 1).

##### Qualitative part

FGD was used as the data collection technique to get participants experiences related to BP and CR. By using open ended guiding list of questions, six sessions of FGDs were undertaken to explore birth plan experience of the community. For urban and rural kebeles, separate FGDs conducted for pregnant women, HDA leaders and fathers. The FGDs were moderated by an experienced health professional and note taker. During discussion, notes were taken and their voices were recorded using tape recorder.

#### Data processing and analysis

##### For quantitative analysis

Collected data were checked for completeness and consistency then coded manually. The coded data were entered into EpiData software version 3.1 then exported in to Statistical Package for the Social Sciences (SPSS) statistics software version 21 to clean, recode and compute statistical analysis. First, descriptive statistics was computed to determine BP and CR variable and to determine the frequency distribution, central tendency and variability of independent variables.

Prevalence of BP and CR was measured by five components such as identified health facility for place of delivery, saved money, decided to deliver by skilled provider, identified mode of transport and arranged blood donor. A pregnant woman was considered as ‘well prepared’ if she spontaneously mentioned at least three BP and CR components otherwise ‘less prepared’. All pregnant women were coded as yes = 1 if well prepared and no = 0 if less prepared.

Attitude and perception variable was measured by using a total of eight questions with five-point Likert scale. The responses were coded as 5 = strongly agree, 4 = agree, 3 = indifferent, 2 = disagree, and 1 = strongly disagree. Responses of all questions were added for each respondent to form composite variable. Negatively worded questions were reverse coded before analysis. Then, mean score was computed to measure the attitudes of participants towards BP and CR. Accordingly, respondents who scored above the mean were considered as having favourable attitude whereas those who scored below the mean were considered as having unfavourable attitude towards BP and CR.

Regarding knowledge of BP and CR, a woman was considered as having “favourable knowledge” if she spontaneously mentioned a total of three obstetric danger signs or more in all the three phases of pregnancy, labour and childbirth and postpartum period otherwise considered as “unfavourable knowledge.”

Wealth index was determined by computing Principal Component Analysis (PCA). Sixteen items were considered for PCA. First, descriptive statistics was used to explore all variables that measure wealth index. Those variables with empty categories and less than 1% observation were excluded from PCA. The resulting household assigned one for asset present and zero for asset absent. Continuous variables were standardized in relation to a standard normal distribution with mean zero and standard deviation one. These standardized scores were then used to create a break points that define wealth index from lowest quintile (poorest) to highest quintile (wealthiest). Each household’s asset for which information collected were assigned a weight or factor score generated through PCA. The PCA result showed that variables highly loaded on the first PCA component were Television, Refrigerator, Electricity and Roof type. Variables highly loaded on the second component were Firewood, Charcoal, finished floor with cement, and wood with cement covered. Variables loaded on the third component were animal drawn cart, horses, and agricultural land. Variables loaded on the fourth component were separate room for kitchen, ownership of home, and Goats.

Independent variables with *p*-value ≤0.25 in bivariate logistic regression analysis were considered as candidate variables for multivariable logistic regression analysis. Those candidate variables were entered into multivariable logistic regression model using backward stepwise method. Hosmer-Lemeshow Goodness-of-fit was checked to test model fitness and the model was best fit (*p*-value =0.402). Multicollinearity were checked using Variance Inflation Factor (VIF) and no significant multicollinearity observed (the largest VIF observed was 1.690). Attitude and perception composite variable was checked for internal consistency with Cronbach’s alpha reliability scale coefficient and showed acceptable scale (=0.735). In multivariable logistic regression analysis, independent variables with *p*-values < 0.05 were reported as significantly associated with dependent variable. The results were presented as frequency table, crude and p-value or adjusted ORs with 95% CI.

##### Qualitative analysis

Prior to analyzing data, all FGDs were transcribed in Amharic text by replaying the recorded voice from tape and the notes taken during discussion. Then, Amharic version text was transcribed in to English language. Different ideas in the text were merged in their thematic areas and a thematic framework analysis was done manually. The results were presented in narration by triangulating with quantitative findings.

#### Data quality management

##### For quantitative study

To ensure the quality of data, the quantitative questionnaire originally prepared in English language were translated to Amharic and Agnua languages by the translators. Those Amharic and Agnua version questionnaire were back translated to English language by another translators. Consistency of the two English version questionnaire were compared by language experts and found consistent. Two days training was provided to data collectors and supervisors on the objectives of the study, data collection tools, data collection procedures and ethical considerations. Moreover, supervisors were trained on supervisory skill about data completeness and cross-checking. To ensure competence of data collectors, peer interviews were made. “Peer interview” in this study used for interviews made between data collectors and supervisors whether they clearly read the questions and ask genuinely in quantitative study. During the peer interview, they exercised to wait for spontaneous responses. The questionnaire were pre-tested on 5% of sample size in Itang District which was not included in the study and analysis. Modifications were made after pretest for skipping pattern, logical sequence of questionnaire sections, choices of some variables. Every collected data were reviewed and checked for completeness and consistency by supervisors within two days of collection.

##### For qualitative study

Open ended guiding questions which originally prepared in English language were translated in to Amharic language text by one person. Following this, Amharic version was back translated in to English by another person to check consistency. Then, the two English versions were compared and found consistent. All FGDs were conducted in Amharic language because all discussants were fluent speakers. Audio-recording tape was used during FGD so that important messages were not missed. A person with an experience of note taking during FGD was assigned for observation and note taking. The FGDs were conducted in the health posts and in the compound of kebele administrative office as the discussants preference.

### Ethical considerations

Ethical clearance was obtained from Institutional Review Board (IRB), Institute of Health, Jimma University. A formal letter from Jimma University was submitted to Agnuak Zone Health Department and respective districts. All pregnant women who fulfill the inclusion criteria for quantitative study were presented with the objectives and rationale of the study. They were also informed about the right to stop the interview at any time if they wished, without giving any reason. Moreover, all data collectors were discussed the issue of confidentiality and obtain verbal consent before the actual interview were launched. For the confidentiality of the information they provided, their names were not written on the questionnaire. For the qualitative study, the moderator explained about the purpose of the study. Following this, voluntary verbal informed consent was obtained from each participants prior to discussion, including to take note and to use tape recorder.

## Results

### Socio-economic and demographic characteristics

A total pregnant women of 403 from urban and 200 from rural area were participated in the study with a response rate of 97.3% in the quantitative study. The mean (± SD) age of pregnant women in urban and rural area were 25.1 (± 4.4) and 26.6(±4.9) years respectively. Regarding their ethnicity, 164(40.9%) from urban and 131(65.5%) from rural were Agnuak. Out of the total respondents, 271 (44.9%) were protestants among which 213(53.3%) urban and 58(29.3%) rural residents (Table 1).

**Table 1:** Socio-economic and demographic characteristics of women by Residence, Agnuak Zone (*n* = 603)

Variables	Urban (*n* = 403)		Rural (*n* = 200)		Total (*n* = 603)		*χ*^2^	*P*-value
	N	%	N	%	N	%		
Age (years)							4.02	< 0.05
< 25	177	44.3	71	35.7	248	41.4		
≥ 25	223	35.8	128	64.3	351	58.6		
Family size							96.44	< 0.001
< 4	205	50.9	36	18.0	241	39.9		
5–6	166	41.2	93	46.5	259	42.9		
≥ 7	32	7.9	71	35.5	103	17.1		
Ethnicity							31.95	< 0.001
Agnuak	165	41.0	131	65.5	296	48.9		
Others^a^	237	59.0	69	34.5	306	50.8		
Religion							52.96	< 0.001
Protestant	213	53.3	58	29.3	271	44.9		
Orthodox	91	22.8	34	17.2	125	20.7		
Catholic	48	12.0	52	26.3	100	16.6		
Others^b^	48	12.0	54	27.3	102	16.9		
Marital Status							3.13	0.077
Married/Cohabited	369	91.6	191	95.5	560	92.9		
Others^c^	34	8.4	9	4.5	43	7.1		
Women’s Educational Status							34.1	< 0.001
No formal education	40	9.9	57	28.5	153	25.4		
Formal education	363	90.1	143	71.5	103	17.1		
Women’s occupational status							13.17	< 0.001
Housewives	267	66.3	161	80.5	428	71.0		
Others^d^	136	33.7	39	19.5	175	29.0		
Husband’s educational status							14.1	< 0.001
No formal education	22	5.7	84	44.4	106	18.4		
Primary school	184	47.7	78	41.3	262	45.6		
Secondary and above	180	46.6	27	14.3	207	36.0		
Husband’s occupational status							21.79	< 0.001
Farmer	77	19.9	156	82.5	233	40.5		
Gov’t/NGO/Self employee	130	33.7	20	10.6	150	26.1		
Merchant	120	31.1	3	1.6	123	21.4		
Others^e^	59	15.3	10	5.3	69	12.0		
Time taken to nearby health institution on foot							442.9	< 0.001
< 1 h	396	98.3	31	15.5	427	70.8		
≥ 1 h	7	1.7	169	84.5	176	29.2		
Wealth quintile							0.054	1.000
First quintile (poorest)	80	19.9	40	20.0	120	19.9		
Second quintile	81	20.1	41	20.5	122	20.2		
Third quintile	77	19.1	39	19.5	116	19.2		
Fourth quintile	85	21.1	41	20.5	126	20.9		
Fifth quintile (wealthiest)	80	19.9	39	19.5	119	19.7		

^a^Oromo, Kambata, Amhara, Tigre, Guraghe, ^b^Islam, Traditional, ^c^Widowed, Single, Separated, Divorsed, ^d^Gov’t/NGO/Self employee, Farmer, Merchant, Student, House maid, Daily laborer, ^e^Daily laborer, Student

Majority of the pregnant women were married [366(90.8%) from urban and 191(95.5%) from rural area]. Regarding their educational status, 65(16.1%) from urban and 88(44.0%) from rural area had no formal education (*p* < 0.001). Twenty two (5.5%) and 84 (42.2%) of their husbands had no formal education from urban and rural area respectively (*p* < 0.001). Occupational status of pregnant women in urban area were statistically different from rural area (*p* < 0.001). Four hundred twenty seven (70.8%) respondents were housewives among which 267(66.3%) were from urban and 160(80.0%) were from rural area. Housewive in this study indicates a married woman who manages the household as her main occupation and whose spouse usually earns the family. Seventy (19.7%) and 154(81.5%) of their husbands from urban and rural area were farmers respectively. Besides, 396(98.3%) from urban and 31(15.5%) from rural resided pregnant women were located in a resident that takes less than one hour travel time on foot to nearby health institution (*p* < 0.001). In terms of their income status, 120 (19.9%) were in the first quintile (poorest) of wealth quintile (Table 1).

### Maternal l characteristics

Significantly higher proportion of respondents from urban area were expecting their first child [138(34.2%) urban and 43(21.5%) rural; *p* < 0.01]. One hundred ninety (74.5%) and 96(61.5%) respondents gave live birth for the first time in urban and rural area respectively (Table 2).

**Table 2:** Obstetric characteristics of respondents by residential area, Agnuak zone (*n* = 603)

Variables	Urban (403)		Rural (200)		Total (603)		*χ*^2^	*P*-value
	N	%	N	%	N	%		
Gravidity							10.76	< 0.01
1	138	34.2	43	21.5	181	30.0		
2–4	232	57.6	134	67.0	366	60.7		
≥ 5	33	8.2	23	11.5	56	9.3		
Parity							822	0.474
1	190	74.5	96	61.5	286	47.4		
2–4	50	19.6	49	31.4	99	16.4		
≥5	15	5.9	11	7.1	26	4.3		
Started ANC service							6.3	0.481
Yes	346	85.9	143	71.5	489	81.1		
No	57	14.1	57	28.5	114	18.9		
Trimester of first ANC visit (by weeks)							97.0	0.476
≤ 12	63	18.3	30	21.3	93	15.4		
13–24	270	78.5	100	70.9	370	61.4		
≥ 25	11	3.2	11	7.8	22	3.7		
Number of ANC visits							11.96	0.478
Planned not to attend at all	24	6.0	38	19.0	62	10.3		
1–3	8	2.0	18	9.0	26	4.3		
≥ 4	366	92.0	144	72.0	510	84.6		
History of obstetric complication							3.19	0.074
Yes	42	10.4	31	15.5	73	12.1		
No	360	89.6	169	84.5	529	87.9		
Decision maker for obstetric care seeking							8.28	0.041
Herself and husband	340	84.4	161	80.5	501	83.1		
Herself only	30	7.4	18	9.0	48	7.9		
Husband only	24	6.0	8	4.0	32	5.3		
Family/relative	9	2.2	13	6.5	22	3.6		

Regarding their ANC visit status, 346(85.9%) and 143(71.5%) pregnant women from urban and rural area had visited at least once during current pregnancy respectively. However, only 93(15.4%) pregnant women started ANC visit during the first twelve weeks of pregnancy in the study area (Table 2).

Forty two (10.4%) urban and 31(15.5%) rural area pregnant women had history of obstetric complication. For a pregnant woman to seek obstetric care, decision maker was significantly different in urban and rural area. Thus, higher proportion of pregnant women from urban area, 340(84.4%) made decision jointly by herself and husband compared to their rural counterparts, 161(80.5%) (Table 2).

Majority of participants in the FGD raised that a pregnant woman started ANC visit to nearby health institute when her pregnancy was about 5 months. The discussants mentioned the importance visiting health institution for ANC service. They said “it is important to attend ANC; to ascertain the duration of the pregnancy, to get vaccinated, to undergo some medical investigations, to know the date of birth, and obtain some medications such as for anemia.”

The participators of FGD were raised the importance of ANC for the health of both woman and her fetus as follows;

*“Yes it is important to follow ANC visit. When I attended a health center, the professional did some checkups and advised me to come back for any unusual signs/symptoms. He also told me to prepare some clothes and bed sheets.”* (A pregnant woman whose age was between 20 and 25; from urban area, FGD5)

*“It is important to follow ANC visit. Because when a woman visited the health center, the doctor might update her on the wellbeing of baby. Besides, she might be informed to make some savings for any uncertainties and to prepare some clothes for the newborn baby.”* A woman whose age was between 30-55; urban HDA, FGD5)

The qualitative finding also pointed out that majority of pregnant women made decisions jointly with their husbands to seek obstetric health care. However, they added that husbands were the usual finance providers especially in rural area; so decisions were not simply by pregnant women. One of the discussant from rural area explained as below;

*“When it comes to decision, husband and wife decide in cooperation. However, a better idea is suggested by the husband. There is occasions she attends by herself, and sometimes, the husband accompanies her. However, I also know husbands who are very strict and never let her to go health institution unless he is willing.”*(A father whose age was between 30-35; from rural area, FGD7)

*“I am the one in charge of offering advice (permission). But, she can still go without my advice or permission since she can do things willfully. I also provide cash when she buys groceries or other necessary materials for her birth related costs.”* (A father whose age was between 30-35; from rural area, FGD6)

The characteristics of FGD participants are provided in the Table 3 below.

**Table 3:** Characteristics of Focus Group Discussion participants by residential area, Agnuak Zone (No of FGD = 6)

Characteristics	Abaru Kebele		Mender 8 and 9 Kebele	
	N	%	N	%
FGD Target Group				
Pregnant Women				
Age in years:				
20–25	5	56	4	44
26–30	4	44	5	56
Marital Status: Married	9	100	9	100
Education level: Read and write			2	22
Primary cycle School (1–4)	2	22	4	44
Secondary cycle School (5–8)	4	45	3	33
High School (9–10)	3	33		
Main Occupation: Housewive/Unemployed	7	78	8	89
Farmer			1	11
Self-employed/Small business	2	22		
HDA Leaders				
Age in years:				
30–35	5	56	2	22
35–40	2	22	3	33
40–45	2	22	4	44
Marital Status: Married	9	100	9	100
Education level: Read and write	1	11	3	33
Primary Cycle School (1–4)	3	33	3	33
Secondary Cycle School (5–8)	3	33	3	33
High School (9–10)	2	23		
Main Occupation: Housewive/Unemployed	7	78	9	100
Self-employed/Small business	2	22		
Farmer				
Fathers				
Age in years:				
30–35	3	33	3	33
35–40	4	45	7	67
40–45	2	22		
Marital Status: Married	9	100	9	100
Education level: Read and write				
Primary Cycle School (1–4)			3	33
Secondary Cycle School (5–8)	4	45	4	45
High School (9–10)	5	56	2	22
Main Occupation:				
Farmer	2	22	7	78
Self-employed/Small business	7	78	2	22

### Knowledge of key danger signs and attitude towards BP and CR practice

Vaginal bleeding was the most common type of a key danger sign spontaneously identified by the pregnant women during pregnancy, childbirth and the postpartum period. However, significantly higher proportion of pregnant women from urban area spontaneously identified it during pregnancy, 135(33.5%); labour and childbirth, 277(68.7%); and postpartum, 221(54.8%) than their rural counterparts; [*p* < 0.001] (Table 4).

**Table 4:** Knowledge of key danger signs and attitude towards birth preparedness and complication readiness of respondents by residential area, Agnuak zone (*n* = 603)

Variables	Urban (403)		Rural (200)		Total		*χ*^2^	*P*-value
	N	%	N	%	N	%		
Knowledge of key danger signs during pregnancy (Multiple responses)								
Severe vaginal bleeding	135	33.5	53	26.5	188	31.2	29.35	< 0.001
Swollen hands/face	38	9.4	4	2.0	42	6.9	14.52	< 0.01
Blurred vision	13	3.2	45	22.5	58	9.6	23.37	< 0.001
Knowledge of key danger signs during labour and delivery (Multiple responses)								
Severe vaginal bleeding	277	68.7	79	39.5	356	59.0	48	< 0.001
Convulsions	23	5.7	4	2.0	27	4.5	8.20	< 0.05
Prolonged labour	156	38.7	60	30.0	216	35.8	7.77	< 0.05
Retained placenta	40	9.9	20	10.0	60	9.9	35.14	< 0.001
Knowledge of key danger signs during postnatal period (Multiple responses)								
Severe vaginal bleeding	221	54.8	65	32.5	286	47.4	32.47	< 0.001
Foul smelling vaginal discharge	10	2.5	16	8.0	26	4.3	19.79	< 0.001
High fever	95	23.6	40	20.0	135	22.4	28.39	< 0.001
Knowledge of key danger signs during pregnancy, labour and delivery, and postpartum							7.16	< 0.01
Favourable knowledge	131	32.5	44	22.0	175	29.0		
Unfavourable knowledge	272	67.5	156	78.0	428	71.0		
Attitude towards birth preparedness and complication readiness						24.44		< 0.001
Favourable attitude	249	61.8	81	40.5	330	54.7		
Unfavourable attitude	154	38.2	119	59.5	273	45.3		

In the study area, only 175(20.0%) pregnant women had favourable knowledge. From these, 134(32%) were from urban area; [*p* < 0.01] (Table 4).

Regarding attitude towards BP and CR practice, significantly higher proportion of pregnant women from urban area, 249(61.8%) had favourable attitude; [*p* < 0.001] (Table 4).

In qualitative study, the most frequently and correctly mentioned danger signs during discussion were vaginal bleeding, prolonged labor, swelling of legs and retained placenta. However, majority of participants mentioned that most of these problems were frequently occur during labour and childbirth or after childbirth. They also mistakenly mentioned anemia, lack of vitamins, loss of appetite, and vomiting as danger signs. A pregnant woman whose age was between 20 and 25 shared the danger signs raised by majority of discussants and explained her idea as written below;

***“****In my opinion, the problem occurs from the beginning of her pregnancy. For example, if she falls down or carried heavy weights, she might experience physical deterioration, running liquids (leakages) in her vagina or change in her voice. At this time, her husband notices and take her to health center. Hence, such experience is not only during childbirth but also throughout her pregnancy.”* (From urban, FGD2)

### Knowledge of community resources

Community services have an important role in reducing barriers of reaching health care contact as well as improving access to a health care facility by the woman during maternal services. However, majority of discussants did not know community support systems. This might be due to one or more reasons such as unavailability of the services, not informed on those available services or difficult to access them because these services are found far away from their residence. For example, majority of discussants were not aware of organized community support services such as a blood bank and ambulance service. In the discussion, they reported that there are communities that a pregnant woman supported by her relatives and sometimes by her neighborhood. The discussants also raised that there are communities that established local group called ‘*edir*’ to support families who are members of the group during difficult condition. Edir is a local voluntary association established by members of the kebele to provide—among others—financial support at the time of emergency medical treatment or referral.

Participants mentioned that a woman get supported by herself or her relatives in emergency situation if she was not a member of the ‘*edir.*’ Bringing clothes and flour, and performing household tasks such as cooking, searching for firewood, and fetching water were the most commonly mentioned community support by discussants. (A pregnant woman whose age was between 26 and 30; from urban area, expressed the issue as follows; FGD2);

*“In my area, there is a traditional association ‘edir’ which established to save money thus a pregnant woman get supported at the time of difficulties.”*

Contrary, another discussant from rural area mentioned;

*“There is no any financial support group in our community. A pregnant woman accompanied by her families or herself whenever she was referred to Gambella or elsewhere.”* (A pregnant woman whose age was between 26-30, FGD3)

Another father from urban area explained his experience as follows:

*“When my wife was referred to Gambella hospital last year, I got cash contributed from myself and my wife’s relatives and we rented a car and went to there.”* (Whose age was between 30-35, FGD4)

*“… There is no blood bank around. It was in Gambella hospital when my wife was in need of blood transfusion... Then, she got transfusion blood from blood bank and then, referred to Jimma referral hospital.”* (A father whose age was between 30-35; from rural area, FGD2)

### Birth preparedness and complication readiness practice by residence

A total of five spontaneous responses of BP and CR practices considered in this study. Among these, significantly higher proportion of urban pregnant women identified a health facility for childbirth and emergency occasions compared to their rural counterparts [183(45.5%) urban and 59(29.5%) rural; *p* < 0.001]. Contrary, significantly higher proportion of pregnant women from rural area identified mode of transport to go health facility for childbirth as well as whenever emergency occurs [66(16.4%) urban and 53(26.5%) rural; *p* < 0.01]. Two hundred seventy five (68.4%) urban and 124(62.0%) rural women saved money to pay for costs related to childbirth and emergency occasions; *p* > 0.05. Fifty two (12.9%) urban and 20(10.0%) rural women identified skilled health personnel who assist them during child birth and emergency occasions. Out of 59(9.8%) pregnant women identified potential blood donor, 44(10.9%) were from urban and 15(7.5%) were from rural area.

The overall prevalence of BP and CR in this study was 23.4%. Significantly higher proportion of pregnant women from urban area spontaneously mentioned at least three BP and CR components than their rural counterparts [104(25.8%) urban and 37(18.5%) rural; *p* < 0.05].

In the qualitative study, majority of discussants mentioned about readiness for childbirth mainly on household preparations related to food, drinks and clothes. They were unable to mention beyond saving money and did not well understood birth preparation components as per the recommendation. The most commonly mentioned items by discussants were food stuffs like butter, drinks like soup and juices, and clothes for newborn and mother. A thirty-two years old father from urban area described the situation as (FGD4);

*“…When my wife was near to give birth, I prepared many things. The surrounding environment were cleaned, I saved money for some expenditures, like to buy bed sheets and groceries. When a woman give birth, there is bleeding, so she needs to eat good diets.”*

Another discussant from rural area described similar idea;

*“…I have prepared butter and bought clothes for myself and my baby. I also saved money.”* (A pregnant woman whose age was between 26-30; from rural, FGD2)

Majority of discussants from urban area raised that major means of transportation in their area were private vehicle and bajaj (a small vehicle which is tricycle). Whereas discussants from rural area mentioned that means of transport mostly available were motorbikes or bajaj. “Even if they saved money to pay for the cost of transportation, the vehicle was not easily available in our area,” majority of discussants said. One of the discussant whose age was between 35 and 40; father from rural area, FGD4 explained as;

*“…There was a man whose wife was told to go to Hospital and wait there until she give birth. But, he ignored and kept her at home. …When the labour started, we had to carry her by local stretcher made of wood to the place where we found small vehicle,* a distance which takes about 20 minutes on foot*. We rented a Bajaj and went to Abobo. When we arrived at Abobo town, we got a car to Gambella town. In such situations, prior readiness is very important. Therefore, lack of preparedness contested us this much.”*

Factors associated with birth preparedness and complication readiness.

From bi-variate logistic regression analysis, factors such as residence, family size, mother’s educational status, mother’s occupational status, husband’s educational status, husband’s occupational status, wealth quintiles, gravidity, trimester of first ANC visit, number of ANC visits, history of obstetric complication, decision maker for obstetric care seeking, time taken to nearby health institution, knowledge status of obstetric danger signs, attitude of women towards BP and CR practice have *p*-value ≤0.25 and considered for multivariable logistic regression.

In multivariable logistic regression analysis, independent predictors of BP and CR with *p*-value < 0.05 were residence, occupational status of pregnant women, history of at least one obstetric complication, trimester of first ANC visit, number of ANC visits, knowledge of three obstetric danger signs at least one from each phase of pregnancy, labour and delivery, and postpartum, attitude of pregnant women towards BP and CR practice and wealth quintile.

Among socio-demographic variables; residence, occupational status of pregnant women and wealth quintiles were found to be associated with BP and CR. Mothers from urban area were about 1.5 times more likely well prepared for birth and its complication than those from rural area (AOR = 1.4; CI: 1.1, 3.8). Mothers having occupation of student were 1.5 times (AOR = 1.5; CI: 1.1, 2.9) and Government employee were about 2 times (AOR = 2.1; CI: 1.3, 5.9) more likely to be prepared for birth and its complication than being housewive. Mothers in the lowest quintile of wealth status (poorest) were about 80% (AOR = 0.2; CI: 0.1, 0.7), in the 2nd quintile were about 70% (AOR = 0.3; CI: 0.1, 0.7) or 3rd quintile were about 60% (AOR = 0.4; CI: 0.2, 0.9) times less likely to be prepared for birth and its complication than those in the fifth quintiles of better wealth status (Table 5).

**Table 5:** Factors independently associated with birth preparedness and complication readiness of respondents in Agnuak zone (*n* = 603)

Variables	BP and CR			Crude OR (95% CI)	Adjusted OR (95% CI)
	Well prepared [N (%)]	Less prepared [N (%)]	Total [N (%)]		
Residence					
Urban	104 (73.8)	299 (64.7%)	403 (66.8)	1.5 (1.1, 2.3)^c^	**1.4 (1.1, 3.8)**^f^
Rural	37 (26.2)	163 (35.3)	200 (33.2)	1	1
Women’s occupational status					
Housewives	75 (53.2)	352 (76.2)	427 (70.8)	1	1
Student	14 (9.9)	57 (12.3)	71 (11.8)	1.9 (1.2, 3.9)^c^	**1.5 (1.1, 2.9)**^f^
Gov’t/NGO/Self employee	23 (16.3)	19 (4.1)	42 (7.0)	5.7 (2.9, 10.9)^c^	**2.1 (1.3, 5.9)**^f^
Merchant	16 (11.3)	22 (4.8)	38 (6.3)	3.4 (1.7, 6.8)^c^	2.9(.9, 9.0)
Other^a^	3 (9.2)	22 (2.6)	25 (4.1)	.6 (0.1, 1.6)^c^	.4(.4, 2.2)
Trimester of first ANC visit					
First trimester	55 (40.4)	38 (10.9)	93 (19.2)	5.6 (3.4, 8.9)^c^	**3.7 (1.8, 7.6)**^d^
Other^b^	81 (59.6)	311 (89.1)	392 (80.8)	1	1
Number of antenatal care visits					
≥ 4 visits	128 (91.4)	261 (57.2)	389 (65.3)	7.9 (4.3, 14.8)^c^	**1.9 (1.2, 4.3)**^f^
< 4 visits	12 (8.6)	195 (42.8)	207 (34.7)	1	1
History of obstetric complication					
Yes	50 (35.5)	23 (5.0)	73 (12.1)	10.5 (6.1, 18.0)^c^	**7.3 (3.1, 17.4)**^d^
No	91 (64.5)	438 (95.0)	529 (87.9)	1	1
Knowledge status of obstetric danger signs					
Favourable knowledge	98 (69.5)	77 (16.7)	175 (29.0)	11.4 (7.3, 17.6)^c^	**6.4 (3.6, 11.4)**^d^
Unfavourable knowledge	43 (30.5)	385 (83.3)	428 (71.0)	1	1
Attitude of women towards BP and CR					
Favourable attitude	117 (83.0)	213 (46.1)	330 (54.7)	5.7 (3.5, 9.2)^c^	**2.3 (1.2, 4.4)**^f^
Unfavourable attitude	24 (17.0)	249 (53.9)	273 (45.3)	1	1
Wealth quintile					
1st quintile (poorest)	16 (11.3)	104 (22.5)	120 (19.9)	0.2 (0.1, 0.4)^c^	**0.2 (0.1, 0.7)**^e^
2nd quintile	26 (18.4)	96 (20.8)	122 (20.2)	0.4 (0.2, 0.6)^c^	**0.3 (0.1, 0.7)**^e^
3rd quintile	23 (16.3)	93 (20.1)	116 (19.2)	0.3 (0.2, 0.6)^c^	**0.4 (0.2, 0.9)**^f^
4th quintile	25 (17.7)	101 (21.9)	126 (20.9)	0.3 (0.2, 0.6)^c^	0.4 (0.2, 1.1)
5^th^quintile (wealthiest)	51 (36.2)	68 (14.7)	119 (19.7)	1	1

^a^Farmer, Daily laborer, house maid, ^b^2nd trimester, 3rd trimester, ^c^*p*-value ≤0.25 and candidate for multivariable logistic regression, ^d^*p*-value< 0.001, ^e^*p*-value < 0.01, and ^f^*p* value < 0.05 in multivariable regression analysis

All entries with boldface are significant

Among the obstetric characteristics; history of obstetric complication, trimester of first ANC visit and number of ANC visits showed significant association. Mothers who had history of obstetric complication found to be about 7 times more likely to be well prepared for birth and ready for complication than their counterparts (AOR = 7.3; CI: 3.1,17.4). Mothers who started ANC visit within 12 weeks after they become pregnant were about 3.7 times more likely to be well prepared than those who started after 12 weeks (AOR = 3.7; CI: 1.8, 7.6). Those mothers who planned to attended four or more ANC visits were about 1.9 times more likely to be well prepared as compared to their counter parts (AOR = 1.9;CI:1.2,4.3) (Table 5).

Knowledge of obstetric danger signs was found to be associated with BP and CR. Mothers who knew three danger signs at least one from each phase of pregnancy, labour and delivery, and postpartum were about 7.3 times more likely to be well prepared than their counterparts (AOR = 7.3; CI: 3.1, 17.4). Regarding mothers’ attitude towards BP and CR practice, those who have favourable attitude were found to be about 2.3 times more likely well prepared than their counterparts (AOR = 2.3; CI:1.2, 4.4) (Table 5).

## Discussions

Nevertheless, BP and CR is one of the proven and effective health care strategy in preventing maternal mortality especially for countries with prevailing high risk of maternal deaths and inefficient health care system [23] the overall prevalence of BP and CR in this study was 23.4% (CI: 20.1, 27.2%). This figure was consistent with study from Northwest Ethiopia [24] of 26.9% and Jimma zone, Ethiopia [19] of 23.3%. The study result showed that significantly higher proportion of urban respondents were well prepared for birth and ready for complication than their counterparts. About 26 % (25.8%; CI: 21.8, 30.3%) of urban and 18.5% (CI: 13.5, 20.5%) of rural pregnant women were well prepared for birth and ready for complication. The current study result of urban area was consistent with study from Adgrat town, Ethiopia [17] which was 22%. In rural locality, our study was lower than 35% of study done in rural Uganda [20]. The dissimilarity might be due to the fact that respondents from Uganda study were recently delivered women. The urban-rural disparity in the current study might be because urban women were more educated, near to information and health facility compared to rural women.

Knowledge status of key obstetric danger signs were significantly different among urban and rural pregnant women. Those mothers who knew three key danger signs at least one in each phase of pregnancy, labour and child birth, and postpartum were more likely well prepared for child birth and ready for complication than counterparts. Similar finding observed in another study [25, 26]. This might be because a woman who did not know a key danger signs of obstetric complications have less intention to seek care than those who are aware of the risks related to pregnancy and child birth. Moreover, they does not have plan to utilize health service at birth or during emergency. This showed that opportunity to increase their plan for birth and emergency preparation by increasing awareness on obstetric danger signs.

This study revealed that women with history of even a single obstetric complication were more likely to be well prepared than those who did not have history of obstetric complication. This might be the fact that those pregnant women could anticipate serious complications from their previous experiences as supported by study conducted in Adigrat town, Ethiopia [17] and Northwest Ethiopia [27].

In this study, mother’s occupation was significantly associated with BP and CR practice. Housewive mothers were less likely well prepared for birth and ready for complication than those who were Government/NGO/Self employee or merchant. This finding was supported by study done in rural communities of Eastern Ethiopia [25]. The possible reason might be housewive women resides at home and were less likely to be exposed to health information related to birth preparedness than counterparts.

ANC visit during the first trimester and number of ANC visits were also another important factors associated with BP and CR. Evidence suggested that ANC is more effective when received in the earlier times of pregnancy [8, 28]. Current study result showed that starting ANC visit before 12 weeks after a mother becomes pregnant increase likelihood of BP and CR. The result was in line with study done in Tanzania [26] but in contrast with study done in Nigeria [29]. The difference might be less attention given to counsel a mother on components of BP and CR in the early stage of pregnancy during ANC visit in the Nigerian study. Previous studies revealed that women who attended ANC at least four times were more likely to be prepared for birth and its complications compared to those who attended less [26, 27]. Similar result obtained in the current study. However in the current study, only 25.8% of women were well prepared indicating the opportunity to advise a woman on all components of BP and CR practice during ANC visit. Our result indicated that mothers who attended ANC were not advised on all components of BP and CR practice.

Mother’s attitude towards BP and CR practice were important predictor of BP and CR in the current study. Mothers with favorable attitudes were more likely to prepare for birth and ready for complication than those with unfavorable attitude. This study was supported by study done in Jimma zone, Ethiopia [19].

In this study, wealth quintiles were identified as a factor associated with BP and CR. Mothers in the fourth quintile were more likely well prepared than those in the third, second or first quintile. This result goes in line with study conducted in Jimma zone [19]. This might be due to the fact that women in the higher quintile have better opportunity for education, professional occupation and increased health seeking behavior.

## Conclusions

Prevalence of BP and CR was low in urban and rural area, though significantly higher in urban area. In this study, three-fourth of women who planned to attend 4+ ANC visits were not counselled on all components of BP and CR. This indicated that opportunity to counsel a woman on all components of BP and CR practice during first ANC visit and subsequent visits.

Predictors of BP and CR identified in this study were; knowledge of obstetric danger signs, trimester of first ANC visit, history of obstetric complication, number of ANC visits, attitude of mothers towards BP and CR practice, residence, mother’s occupational status and wealth quintile.

All health care providers should counsel every pregnant woman who come to health institution for ANC service on BP and CR components during her first ANC visit and all subsequent visits. Moreover, counselling pregnant women on obstetric danger signs and promoting them on favourable attitude towards BP and CR are recommended to improve BP and CR practice. Community based interventions such as pregnant women conferences are also entry point to provide information and education on obstetric danger signs and components of BP and CR which likely to increase BP and CR practice. Improving educational status of women and promoting them on income generating activities which likely to raise socioeconomic status also increases BP and CR practice. Further research should be done on weather health workers have knowledge gap and/or they did not provide information on the BP and CR.

## Supplementary information

**Additional file 1.** Questionaire (English Version).docx.

## Data Availability

The datasets used and/or analyzed during the current study are available from the corresponding author on reasonable request.

## Abbreviations

ANC: Antenatal Care
BP and CR: Birth Preparedness and Complication Readiness
EDHS: Ethiopia Demographic and Health Survey
FGD: Focus Group Discussion
HDA: Health Development Army
LMIC: Low and Middle Income Countries
PCA: Principal Components Analysis
SRS: Simple Random Sampling
SSA: Sub-Saharan Africa
