# Supplementary material for: Birth preparedness, complication readiness and associated factors among pregnant women in Agnuak zone, Southwest Ethiopia: a community based comparative cross-sectional study
Source: BMC Pregnancy Childbirth. 2020 Feb 3;20:72. doi: 10.1186/s12884-020-2766-9 (PMC6998184; doi:10.1186/s12884-020-2766-9)
Supplement: Supplementary file 1 — Additional file 1. Questionaire (English Version).docx. [file 12884_2020_2766_MOESM1_ESM.docx]

Annex-C: English Version Questionnaires

**JIMMA UNIVERSITY**

**INSTITUTE OF HEALTH SCHOOL OF GRADUATE STUDIES**

**DEPARTMENT OF POPULATION AND FAMILY HEALTH**

***Quantitative Questionnaire on birth preparedness and complication readiness among pregnant women in Agnuak Zone, Gambela Regional State***

**Informed Consent Form for Quantitative Survey**

Good morning /after noon

My name is…………………………………

First of all, I would like to thank you for giving your time.

I am working as data collector with the research team of Jimma University Institute of Health which conducting research on pregnant women from April 10 to May 10, 2017 in Agnuak Zone. The aim of this study is to assess status of birth preparedness and complication readiness among pregnant women. The information obtained from this study by your participation will help you/mothers to prepare for safe delivery, the health care provider for proper service provision, the government for logistic supply and to design evidence based strategy to improve institutional delivery. The study will not cause any harm to you except giving the information. The information will be collected from the above mentioned mothers by using pretested structure questioners on Socio-demographic information, past obstetric history, Knowledge of danger signs, Plan to use obstetric services and Knowledge of Community Resources through house hold in selected kebeles. We are inviting pregnant women with gestational age above 12 weeks to contribute for the study. You have been selected randomly for this interview, so I would like to ask you some questions related to the subject. Your name will not be recorded and all the information you give will be kept strictly confidential and is to be used only for the purpose of this study. You have the right to refuse participation at any time. The interview will take approximately 30 min.

Are you willing to participate?

Yes No

If she say yes, say thanks and proceed to the consent form. If say no, say thanks, do not force or reinforce to participate in the study.

**Consent form**

I have read or it has been read to me in the language I understand about the above stated conditions therefore I am willing to participate in this study.

Signature of participant/fingerprint…………………………….

Date of interview…………………………..

Result of interview

1. Completed 2. Partially completed. 3. Refuse

Name of interviewer…………………….…. Signature………………….

Name of supervisor …………………………Signature………………….

Date checked……………………………… (DD/MM/YY)

In case you need to contact:

Contact address of the investigator: Name: Fikru Letose

Mobile: 0921416858

Email: fikruletose6 @gmail.com

WOMAN IDENTIFICATION NO……….

1. Woreda: ………………….

2. Kebele: ………………..

3. Got: …………………….

4. Gare: …………………….

5. Name of Head of House Hold …………………….

1. SOCIO-DEMOGRAPHIC INFORMATION

| Q.# | Question | Codes | Go to Q |
| --- | --- | --- | --- |
| 101 | Residence of the women? | 1.urban 2.rural |  |
| 102 | How old are you? | ---------------------Years |  |
| 103 | What is your religion? | 1.Orthodox Christian  2.Islam  3.Protestant  4.Catholic  5.Other (specify) ----------------- |  |
| 104 | What is your ethnicity? | 1.Agnuak 4.Tigire  2.Oromo 5.Amhara  3.Kambata 6.Nuer  7.Other (Specify) ----------------- |  |
| 105 | How many is your number of family that lives with you? | ----------------------- |  |
| 106 | What is your marital status now? | 1.Married 4.Separated  2.Cohabited 5.Divorced  3.Single 6.Widowed  7.other(specify) |  |
| 107 | What is your educational status? | 1.Can’t read and write  2.Read and write only  3. Grade 1-4  4. Grade 5-8  5. Grade 9-12  6. College  7. University |  |
| 108 | What is your occupation? | 1.House wife  2.Farmer  3.House maid  4.Gov’t/NGO employee  5.Merchant  6.Student  7.Other(Specify) |  |
| 109 | In addition to your house work, do you do any other work for which you are paid in cash or in kind? | 1.Yes  2.No | If NO, Go to 🡪**Q111** |
| 110 | If yes to Q109, how much birr is paid for you per month? | ____________ET. Birr |  |
| 111 | What is your husband’s/partners educational status? | 1.Can’t read and write  2.Read and write only  3.Grade(1-4)  4.Grade(5-8) 5.Secondary and above  6. I have no partner | Currently, if you have no partner/ not in marital union,  🡪Q114 |
| 112 | What is your Husband’s/partners current occupation? | 1.Farmer  2.Gov’t/NGO employee  3.Merchant  4.Student  5.Daily laborer  6.Other(Specify)____________ |  |
| 113 | What is approximate monthly income of your husband per month? | ___________ET. Birr |  |
| 114 | What is the main source of drinking water for members of your household? | 1.Pipe water  2.Public hand pump  3.Public tap/standpipe(Bono)  4.Protected dug well  5.Unprotected dug well  6.Protected spring  7.Unprotected spring  8.Rain water |  |
| 115 | What kind of toilet facility do you have that your household members use? | 1. Flush toilet  2. Traditional Pit toilet/latrine  3. Ventilated Improved Pit (VIP) latrine  4. No facility-use open field  5.Other(specify)  __________________ |  |
| 116 | How is your residential home ownership? | 1.Own home  2.Rented home  3.Other (specify)________ |  |
| 117 | How many rooms/classes does your household have | _________ rooms |  |
| 118 | How many rooms in this household are used for sleeping | _________ rooms |  |
| 119 | Do you have a separate room which is used as a kitchen? | 1.Yes 2.No |  |
| 120 | What is the main material of the floor of the home?  (RECORD BY OBSERVING) | 1.Natural floor- earth  2.Natural floor-dung  3.Rudimentary floor with  wood/bamboo  4.Finished floor with Cement  5.Other (specify) ______ |  |
| 121 | What is the main material of the roof of the home? (RECORD BY OBSERVING) | 1.Thatched  2.Corrugated iron sheet  3.Other (specify) ________ |  |
| 122 | What is the main material of the wall of the home? (RECORD BY OBSERVING) | 1.No wall  2.Wood without mud  3.Wood with mud  4.Wood with cement covered  5.Cement blocks or Bricks  6.Other (specify) ____________ |  |
| 123 | What type of fuel do you mainly  use for cooking in your  Household? | 1.Electricity 4.Charcoal  2.Biogas 5.Firewood  3.Kerosene 6.Dung  7..Other (specify) ___________ |  |
| 124 | Does your household own the following? | |  |
|  | Electricity? | 1.Yes 2.No |  |
|  | Radio? | 1.Yes 2.No |  |
|  | Television? | 1.Yes 2.No |  |
|  | A landline telephone functioning? | 1.Yes 2.No |  |
|  | Refrigerator? | 1.Yes 2.No |  |
| 125 | Does any member of your household own the following? | |  |
|  | Watch? | 1.Yes 2.No |  |
|  | Mobile phone? | 1.Yes 2.No |  |
|  | Bicycle? | 1.Yes 2.No |  |
|  | Motor cycle? | 1.Yes 2.No |  |
|  | Animal drawn cart? | 1.Yes 2.No |  |
|  | A car or truck | 1.Yes 2.No |  |
| 126 | Does any member of this household own any agricultural land? | 1.Yes 2.No | If NO, Go to  🡪Q128 |
| 127 | If Yes to Q124, how many hectares? | _____________hectares |  |
| 128 | Does this household own any livestock, herds, other farm animals, or poultry? | 1.Yes 2.No | If NO, Go to 🡪Q130 |
| 129 | If yes to Q032, how many: | |  |
|  | 1. Cattle? |  |  |
|  | 2. Milk cows or bulls? |  |  |
|  | 3. Horses, donkeys or mules? |  |  |
|  | 4. Goats? |  |  |
|  | 5. Sheep? |  |  |
|  | 6. Chickens? |  |  |
| 130 | What is the approximate time it takes from the nearest health center on foot (in munities)? | ------------------minute |  |
| 131 | What is the approximate time it takes from the nearest hospital on foot (in munities)? | -------------------minute |  |

**MATERNAL FACTORS**

2. PAST OBSTETRIC HISTORY

| Q. # | Question | Code | Go to Q |
| --- | --- | --- | --- |
| 201 | How many pregnancies have you ever had, including current pregnancy, abortion and stillbirth? | 1.First pregnancy  2.two and above | If this is her 1^st^ pregnancy, skip to  🡪301 |
| 202 | Did any of these pregnancies ended in abortion (termination of pregnancy before 28 weeks of gestation)? | 1.Yes 2.No | If NO, Go to 🡪Q204 |
| 203 | If Yes to Q202, how many of them ended in abortion? | ----------------times |  |
| 204 | Did any of these pregnancies ended in stillbirth (delivery ended in birth of dead foetus after 28 weeks of gestation)? | 1.Yes 2.No | If NO, Go to  🡪Q206 |
| 205 | If Yes to Q204, how many of them ended in still birth? | ----------------times |  |
| 206 | How many of them ended in live birth (a new-born that showed any signs or life)? | ------------------ |  |
| Add The Responses Of Q203,Q205 And Q206 and Compare With Q201 and Reconsider For Discrepancies --------------- | | | |

3. PLAN TO USE OBSTETRIC SERVICES

| Q.# | Question | Code | Go to Q |
| --- | --- | --- | --- |
| 301 | Did you have any Antenatal care during this pregnancy? | 1.Yes 2.No | If NO, Go to 🡪**Q306** |
| 302 | If Yes to Q301, where was the place for ANC? | 1.Hospital  2.Health centre  3.Health Post  4.Home of the respondent  5. Other (Specify)..................... |  |
| 303 | If Yes to Q301, How many times in total did you receive ANC for this pregnancy till today? | -----------------times |  |
| 304 | If Yes to Q301, whom do you see for the ANC? | 1.Doctor  2.Nurse  3.Midwife  4.Health Officer  5.Health Extension Worker  6.Family Member  7.Other (specify _________ |  |
| 305 | If Yes to Q301, at what weeks of Gestation did you have the first care/Visit? | ------------------weeks |  |
| 306 | How many ANC Visits have you planned to attend at all? (the attended[if you attended] and future plan) | 1.Planed not to attend at all  2.Once only  3.Twice only  4.Three times only  5.Four times and above  6. Any other response (Specify)……………….. |  |
| 307 | Who can made the final Decision for your obstetric health care seeking? | 1.Herself only  2.Husband only  3.Herself & husband/partner/family/relative/mother in law  4.Family/relatives |  |
| 308 | Have you planed on the place where to give this birth? | 1.Yes 2.No | If NO, Go to **Q311** |
| 309 | If Yes to Q307, where have you planned to deliver? | 1.Hospital  2.Health centre  3.Health Post  4.Home of the respondent  5. Other (Specify) ....................... |  |
| 310 | Why did you prefer this place for your plan? PROB: Any other reasons?  (More than one answer is possible) | 1.The facility is near to me  2.Gave better service  3.I had better out come before  4.Health workers advice  5.Difficulty of labour  6.I had problem with previous home deliveries  7.Others,specify------------ |  |
| 311 | Have you planned by whom to be attended for the delivery of this pregnancy? | 1.Yes 2.No | If No skip **Q312** & **Q313** |
| 312 | If Yes to Q311, by whom did you plan to be attended? | 1.Doctor  2.Nurse  3.Midwife  4.Health Officer  5.Health extension worker  6.Family Members |  |
| 313 | Why did you prefer this attendant for your plan? PROB: Any other reasons? | ---------------------------------  ---------------------------------- |  |

**KNOWLEDGE RELATED FACTORS**

1. KNOWLEDGE OF DANGER SIGNS

Instruction፡

- From question number 403-405 first read the question only then probe them if additional danger sign they mention.
- Infront of each response on empty box put number “1” when respondents mentioned obstetric danger sign spontaneously, put “2” when respondents answer obstetric danger sign after you mention the items and put :3” when respondent say I don’t know (for Q no 403-405).

| Q.# | Question | Code | | Go to Q |
| --- | --- | --- | --- | --- |
| 401 | In your opinion, can unforeseen problems related to pregnancy, delivery or child birth occur? | 1.Yes 2.No  3. I don’t know……….. | | If NO, Skip 402 |
| 402 | If Yes to Q401, Do You think that these problems threaten the life of the women? | 1.Yes 2.No  3. I Don’t know | |  |
| 403 | What are some of serious health problems that can occur **during pregnancy**?  PROBE: Any others? | 1.Vaginal Bleeding |  |  |
|  |  | 2.Blurred vision |  |  |
|  |  | 3.Swollen hands/face |  |  |
|  |  | 4.Severe headache |  |  |
|  |  | 5.Convulsions |  |  |
|  |  | 6.High fever |  |  |
|  |  | 7.Loss of consciousness |  |  |
|  |  | 8.Difficulty breathing |  |  |
|  |  | 9.Sever weakness |  |  |
|  |  | 10.Severe abdominal pain |  |  |
|  |  | 11.Accelerated/reduced foetal mov’t |  |  |
|  |  | 12.Water breaks without labour (PROM) |  |  |

| 404 | What are some serious health problems that can occur **during labour and child birth** that could endanger the life of the woman?  PROBE: Any others? | 1.Severe Vaginal Bleeding |  |  |
| --- | --- | --- | --- | --- |
|  |  | 2.Blurred vision |  |  |
|  |  | 3.Placenta not delivered 30 minutes after baby |  |  |
|  |  | 4.Severe headache |  |  |
|  |  | 5.Convulsions |  |  |
|  |  | 6.High fever |  |  |
|  |  | 7.Loss of consciousness |  |  |
|  |  | 8.Labour lasting >12 hours |  |  |
| 405 | What are some serious health problems that can occur **during the first 2 days after birth** that could endanger the life of the woman?  PROBE: Any others? | 1.Severe Vaginal Bleeding |  |  |
|  |  | 2.Blurred vision |  |  |
|  |  | 3.Swollen hands/face |  |  |
|  |  | 4.Severe headache |  |  |
|  |  | 5.Convulsions |  |  |
|  |  | 6.High fever |  |  |
|  |  | 7.Loss of consciousness |  |  |
|  |  | 8.Difficulty breathing |  |  |
|  |  | 9.Sever weakness |  |  |
|  |  | 10.Malodorous vaginal discharge |  |  |

5. BP&CR PRACTICE AND KNOWLEDGE OF COMMUNITY RESOURSES

Instruction:

- For question number 502 first read the question and wait for spontaneous response then if no more they able to mention, probe them for additional BP&CR practice.
- Infront of each response on empty box put number “1” when respondents mentioned BP&CR practice spontaneously, put “2” when respondents answer BP&CR practice after you mention the items and put :3” when respondent say I don’t know for Q no 502.

| Q.# | Question | Code | | Go to Q |
| --- | --- | --- | --- | --- |
| 501 | Have you ever heard the term “Birth preparedness”? | 1.Yes 2.No | |  |
| 502 | In your opinion, what are some things a woman can do to prepare for birth?  PROBE: more than one answer is possible so ask as ‘any others? Until she says no more. | 1.Identify mode of transport |  |  |
|  |  | 2.Save money |  |  |
|  |  | 3.Identify blood donor |  |  |
|  |  | 4.Identify place of delivery |  |  |
|  |  | 5.Identify skilled provider |  |  |
|  |  | 6.Identify who accompanies |  |  |
|  |  | 7.Identify decision maker |  |  |
|  |  | 8. Grain for porage/other food item |  |  |
|  |  | 9. Rather blade, thread |  |  |
|  |  | 10. Cloth for new born |  |  |
|  |  | 11..Others (list) _________________ | |  |
| 503 | Does your community provide services to assist women in preparing for birth? For instance: | | |  |
|  | 1. Are there transportation services for woman? | 1.Yes 2.No  I Don’t know………………. | |  |
|  | 2. Are there ways to get money to help families pay for birth? | 1.Yes 2.No  I Don’t know………………. | |  |
|  | 3. Are there ways to get blood donated during pregnancy or complications? | 1.Yes 2.No  I Don’t know | |  |
| 504 | Which one of the above have you/your family planned to arrange? | | |  |
|  | 1. Have you/your family planned to arrange transportation services for this birth? | 1.Yes 2.No | |  |
|  | 1. Have you/your family planned to save money to help you for during this birth? | 1.Yes 2.No | |  |
|  | 1. Have you/your family planned to arrange ways to get blood donation during pregnancy or complications of this birth? | 1.Yes 2.No | |  |

1. ATTITUDES AND PERCEPTIONS ABOUT BP&CR

Now I am going to read out a list of common perceptions about pregnancy, childbirth, and the period immediately after childbirth. I would like to know whether you 1=Strongly disagree(SD), 2=Disagree(D), 3=Indifferent(ID), 4=agree(A), or 5=Strongly agree(SA) with these statements. There is no right or wrong answer to any of these questions. We are only interested in hearing your opinion.

| **Q.#** | **Questions** | **Response Codes** | | | | |
| --- | --- | --- | --- | --- | --- | --- |
|  |  | **SD (1)** | **D (2)** | **ID (3)** | **A**  **(4)** | **SA**  **(5)** |
| 601 | A pregnant woman should plan ahead of time where she will give birth to her baby. |  |  |  |  |  |
| 602 | A pregnant woman should plan ahead of how she will get to the place where she will give birth. |  |  |  |  |  |
| 603 | It is necessary for a husband/partner to accompany his wife to ANC visits. |  |  |  |  |  |
| 604 | It is necessary for a husband/partner to accompany his wife when she is giving birth. |  |  |  |  |  |
| 605 | Giving birth is mostly a woman’s matter. Husbands/partners have nothing to contribute. |  |  |  |  |  |
| 606 | When women do not go to a health facility to give birth, it is mainly because it is too expensive. |  |  |  |  |  |
| 607 | When women do not go to a health facility to give birth, it is mainly because the staff there do not treat women respectfully. |  |  |  |  |  |
| 608 | When women do not go to a health facility to give birth, it is mainly because it is too difficult to get there. |  |  |  |  |  |

I have finished the interview. Thank you for spending your time and valuable information you gave us.
